# Diazotrophs for Lowering Nitrogen Pollution Crises: Looking Deep Into the Roots

**DOI:** 10.3389/fmicb.2021.637815

**Published:** 2021-05-24

**Authors:** Asma Imran, Sughra Hakim, Mohsin Tariq, Muhammad Shoib Nawaz, Iqra Laraib, Umaira Gulzar, Muhammad Kashif Hanif, Muhammad Jawad Siddique, Mahnoor Hayat, Ahmad Fraz, Muhammad Ahmad

**Affiliations:** ^1^Division of Soil and Environmental Biotechnology, National Institute for Biotechnology and Genetic Engineering-Campus-Pakistan Institute of Engineering and Applied Sciences (NIBGE-C-PIEAS), Faisalabad, Pakistan; ^2^Department of Bioinformatics and Biotechnology, Government College University, Faisalabad, Pakistan; ^3^Department of Botany, Government College University, Faisalabad, Pakistan; ^4^Department of Botany, University of Bagh, Kotli, Pakistan; ^5^Department of Biotechnology, Institute of Molecular Biology and Biotechnology, University of Lahore, Sargodha, Pakistan

**Keywords:** nitrogen fixation, rhizobia, slow-releasing fertilizers, nitrogen use efficiency, nitrogen pollution

## Abstract

During and after the green revolution in the last century, agrochemicals especially nitrogen (N) were extensively used. However, it resulted in a remarkable increase in crop yield but drastically reduced soil fertility; increased the production cost, food prices, and carbon footprints; and depleted the fossil reserves with huge penalties to the environment and ecological sustainability. The groundwater, rivers, and oceans are loaded with N excess which is an environmental catastrophe. Nitrogen emissions (e.g., ammonia, nitrogen oxide, nitrous oxide) play an important role in global climate change and contribute to particulate matter and acid rain causing respiratory problems, cancers, and damage to forests and buildings. Therefore, the nitrogen-polluted planet Earth needs concerted global efforts to avoid the disaster. Improved agricultural N management focuses on the synchronization of crop N demand and N supply along with improving the N-use efficiency of the crops. However, there is very little focus on the natural sources of N available for plants in the form of diazotrophic bacteria present inside or on the root surface and the rhizosphere. These diazotrophs are the mini-nitrogen factories that convert available (78%) atmospheric N_2_ to ammonia through a process known as “biological nitrogen fixation” which is then taken up by the plants for its metabolic functioning. Diazotrophs also stimulate root architecture by producing plant hormones and hence improve the plant’s overall ability to uptake nutrients and water. In recent years, nanotechnology has revolutionized the whole agri-industry by introducing nano-fertilizers and coated/slow-releasing fertilizers. With this in mind, we tried to explore the following questions: To what extent can the crop N requirements be met by diazotroph inoculation? Can N input to agriculture be managed in a way leading to environmental benefits and farmers saving money? Can nanotechnology help in technological advancement of diazotroph application? The review suggests that an integrated technology based on slow-releasing nano-fertilizer combined with diazotrophs should be adopted to decrease nitrogen inputs to the agricultural system. This integrated technology would minimize N pollution and N losses to much extent.

**GRAPHICAL ABSTRACT:**
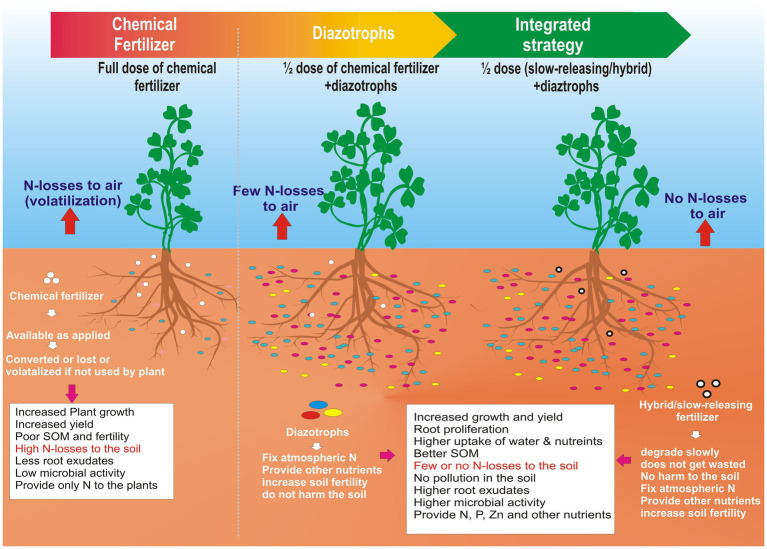
A road map to lowering nitrogen pollution in the atmosphere.

## Nitrogen Fertilizer: Trends, Use Efficiency, and Problems of Excessive Usage

Nitrogen (N) is a vital element for life. Apart from being an integral component of DNA, it is part of amino acid, NAD, and ATP in the cells of living organisms. The nitrogen available for plant uptake is the key determining factor for plant yield as it plays an important role in plant metabolism and food quality (Massignam et al., 2009). N deficiency in plants leads to pale yellowish-green plants with slow growth, smaller flowers and dormant buds, and reduced tiller and fruit development. The N moves from organic form (plant materials) to inorganic form in soil upon decomposition, and in this way, soil fertility is also maintained (Dong et al., 2015).

**Figure 1:**
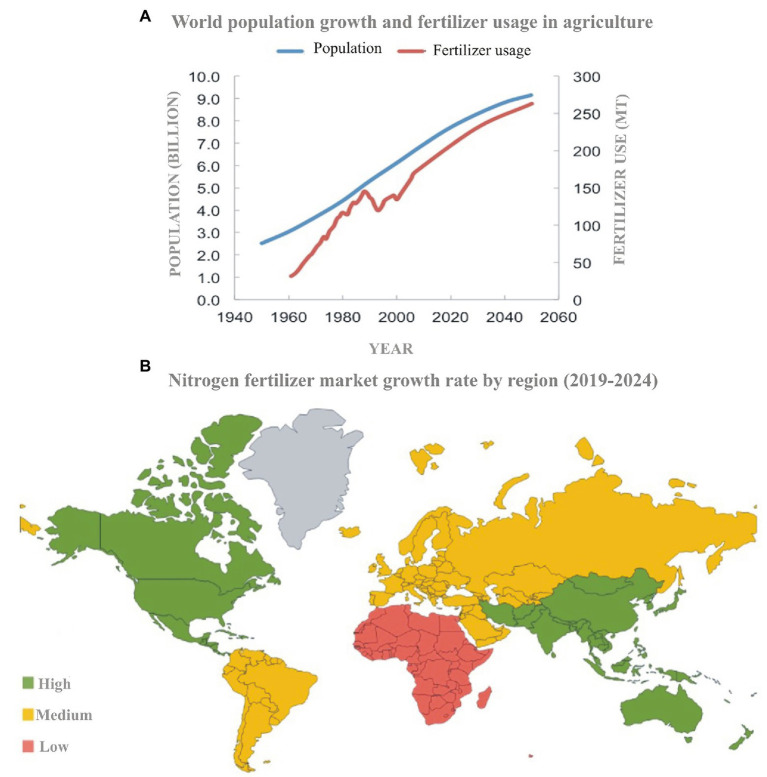
Fertilizer usage and population growth trends in the last 100 years and future prediction (source: Meer, 2016; **A**). Map showing the region-wise N-fertilizer input into the agricultural system (source: Mordor Intelligence; **B**).

The global N cycle has been altered more than that of any other basic element intentionally by using it as a fertilizer or unintentionally as a by-product of fossil fuel combustion. The global population directly or indirectly relies on N fertilizer for food supply. The fertilizer trends (Figure 1A) show a synchronized growth of the population and N usage. The 2nd half of the 20th century showed an 80% increase in N usage from 11 million tons in 1961 to 119 million metric tons in 2018 (Liu et al., 2015; FAO, 2017). The Asia-Pacific region is the largest consumer of the total N-fertilizer market (Figure 1B), accounting for 60% of the total global N-fertilizer usage. Although N fertilizer is necessary for most of the crops, yields have nearly reached their biological maxima whereas farmers keep on adding more and more fertilizers. Overall, N added to the field is about 10-fold higher than it is consumed (Erisman et al., 2013) because crops have low N uptake (30–40%) and thus low N-use efficiency (20–40%; Zhu et al., 2016; Omara et al., 2019). Excess fertilizer does not become part of the plant, but it leaches down and becomes part of the soil or is emitted to the atmosphere (Sharma and Bali, 2018). This shows that agriculture is the primary source of about two-thirds of global N pollution.

Nitrogen emissions (e.g., ammonia, nitrogen oxide, nitrous oxide) play an important role in global climate change and contribute to particulate matter and acid rain causing respiratory problems, cancers, and damage to forests and buildings. Nitrous oxide (N_2_O) is a potent greenhouse gas, over 300 times more effective in trapping heat than carbon dioxide, and remains in the atmosphere for ≈114 years (Good and Beatty, 2011). Excessive N accumulation alters the atmospheric concentration of three anthropogenic greenhouse gases CO_2_, CH_4_, and N_2_O, and plays a crucial role in climate change. Mitigation of N use can lower the atmospheric burden of these greenhouse gases, thereby slowing the rate of climate change over the coming years.

Apart from the climate change, excessive use of N has led to increased pest infestation, e.g., aphids (Nevo and Coll, 2001), and accumulation of heavy metals (e.g., lead, chromium, and cadmium), radionuclides, and carcinogenic compounds (e.g., nitrosamine) as well as accumulation of NO_2_ and NO_3_ (Savci, 2012). Excessive use of N causes serious health concerns related to respiratory ailment, cardiac arrest, and several vector-borne diseases like malaria and cholera in cattle and humans (Ward et al., 2018). In Europe alone, the environmental and human health costs of N pollution are estimated to be 70–320 billion euros per year (Sutton et al., 2011). The continuous enrichment of N in the water leads to excessive growth of algae and other plants, a phenomenon known as “eutrophication” leading to the development of “dead zones.” These dead zones do not support any life forms due to the lack of oxygen and are found in any lake or coastal area. Eutrophication mediates the growth of harmful algal blooms (HABs; Paerl et al., 2011), and water becomes contagious for fisheries and drinking due to the increased growth of algae and oxygen shortage caused by their decomposition. The overall processes of HABs lead to global warming, salinization (drought), and longer seasons affecting plant growths as well. Excessive use of N also disrupts the growth and development of agronomic plants, especially affecting the phenolic, flavonoid, oil, and sugar contents in oil crops (*Sesamum indicum*) as well as antioxidant activity (Elhanafi et al., 2019). The ecological response to elevated nitrogen may increase allergenic pollen production in plants (Reinmuth-Selzle et al., 2017). Moreover, processes like nitrification, mineralization, and ammonia volatilization in air, water, and soil leachate leads to high nitrite nitrogen in drinking water, rivers, and crops, posing potential health hazards to ecosystems (Jia et al., 2015).

Management of N is a challenging goal and needs combined efforts to improve its efficiency. To avoid the problem from worsening, scientists warn that the global N use must cut back and N efficiency be increased in agriculture farms. European countries, being the leaders in “green policies” along with UN Environment Programme through the combination of the best nutrient management practices (BNMPs), International Nitrogen Management System (INMS), advances in fertilizer technology, and plant genetics, collectively aim to reduce worldwide N applications by 20–30% till 2050, saving US$ 150B annually.

## Biological Nitrogen Fixation to Replace Fertilizer N

The process through which atmospheric nitrogen (N_2_) is converted to ammonia (NH_3_) is referred to as the biological nitrogen fixation (BNF), and the nitrogen-fixing bacteria are known as “Diazotrophs” (Shin et al., 2016). The BNF in legumes was described by Hellriegel and Wilfarth (1888) and Franche et al. (2009), and in cyanobacteria by Stewart (1969). The BNF is a highly energy-expensive process that is undertaken by symbiotic bacteria inside the root nodules and in soil or on plant roots by nonsymbiotic bacteria. This process is the natural and major source of nitrogen for plants within a range of ecosystems (Angus and Grace, 2017) and the key contributor to the N economy in the biosphere providing ≈128 Tg of N per annum globally (Vitousek et al., 2013), which is equal to 60% of total fixed N. Under field, diazotrophs fix ≈30–50% of N (Ormeño-Orrillo et al., 2013) contributing up to 15% of the total plant’s nitrogen. A low level of combined nitrogen and a high level of carbon compounds trigger the nitrogen-fixing activity of microorganisms. The process requires 1 mol of N_2_, a supply of electrons and protons, and 16 moles of ATP to produce 2 mol of ammonia as shown in the following equation:

$$N_{2}+16\mathrm{MgATP}+8e^{-}+8H^{+}\to 2{\mathrm{NH}}_{3}+H_{2}+16\mathrm{MgADP}+16\mathrm{Pi}$$

The formation of ammonia from molecular hydrogen and nitrogen has an overall negative enthalpy of reaction (ΔH°= −45.2 KJ mol^−1^ NH_3_); the energy barrier to activation is generally insurmountable (E_A_°= 420 KJ mol^−1^) without the assistance of catalysis. The enzyme, therefore, requires a great deal of chemical energy, released from the hydrolysis of ATP (16 mol of ATP for each mole of N_2_ reduced), and reducing agents, such as dithionite *in vitro* or ferredoxin *in vivo*.

## Plant-Microbe Interaction Concerning BNF and Suitable Modifications to Harness the Potential of BNF

**Figure 2:**
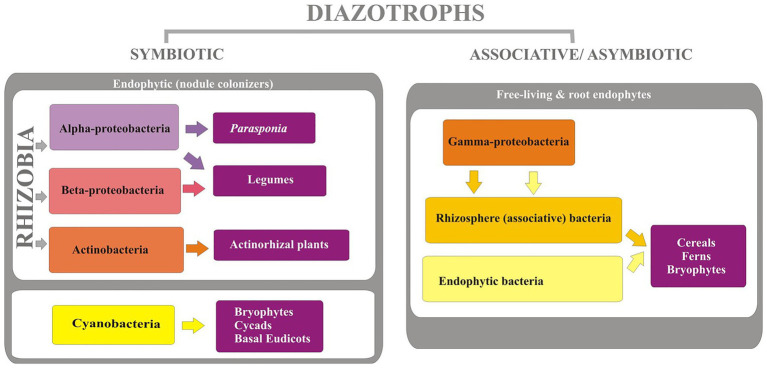
List of different diazotrophs and their hosts.

The diazotrophs are phylogenetically diverse (Figure 2) comprising of organisms of varying physiological properties and belong to taxonomically diverse groups of bacteria from alphaprotobacteria, betaprotobacteria, gammaprotobacteria (Shin et al., 2016). Major genera include *Azospirillum*, *Azorhizobium*, *Pseudomonas*, *Rhizobium*, and phyla cyanobacteria, and Firmicutes. The three major types of bacteria that fix the nitrogen are rhizospheric, endophytic, and nodulating bacteria (Brusamarello-Santos et al., 2012) with a varying range of nitrogen fixation (Table 1). The associative and symbiotic diazotrophs are detailed below.

**Table 1:** Estimated nitrogen fixation rates of diazotrophs.

Microorganism	Types of N fixation	Fixed N transfer	Amount of N-fixation (kg N ha^−1^ Y^−1^)
*Acetobacter*			200
*Azospirillum*			50–465
*Burkholderia*	Associative	High	
*Herbaspirillum*			
Rhizobia and *Frankia*	Symbiotic	Moderate	2–170
Cyanobacteria *Rhodobacter*	Free living	Low	1–80
*Azotobacter*			50

### Associative Diazotrophs

Diazotrophs present in the soil mostly belong to this group and form a subset of the plant growth-promoting rhizobacteria (PGPR) which are directly beneficial for plant growth and yield. They reside on the surface of roots specifically in the zone of elongation or in root hairs. The bacteria found inside the shoot/root of the phyllosphere of the plants are classified as endophytic diazotrophs, and those found on the root surface are associative diazotrophs (Bashan et al., 2004). In a relative comparison of plant growth promotion and nitrogen assimilation by the plants, endophytic diazotrophs have an advantage over associative diazotrophs due to the establishment of better and stable niche within the plant tissues for nitrogen fixation and further assimilation of fixed nitrogen compounds by the plants (Moyes et al., 2016). The presence of these diazotrophs in the proximity to roots helps in the nutrient acquisition of plants, ultimately influencing the growth and yield in various crops (Wani et al., 2013).

They are omnipresent and belong to different genera, such as *Cupriavidus*, *Frankia*, sulfate-reducing bacteria, *Pseudomonas*, *Azoarcus*, *Azospirillum*, *Burkholderia*, *Azorhizobium*, *Gluconacetobacter*, *Citrobacter*, *Klebsiella*, *Enterobacter*, and *Herbaspirillum* (Santi et al., 2013). Examples are *Herbaspirillum* and *Burkholderia* which fix nitrogen in vesicular and intercellular spaces of sugarcane (Van Deynze et al., 2018), and *Azospirillum*, which is a facultative endophytic diazotroph of cereals. They move with the help of flagella to migrate to a micro-aerophilic condition where the nitrogenase enzyme is protected from oxygen (Steenhoudt and Vanderleyden, 2000). *Azospirillum* frequently exists in different regions of the world and develops an association with a wide-ranging species of plants (Pereg et al., 2016).

### Symbiotic Diazotrophs

One of the best-studied symbioses is the symbiotic nitrogen fixation that involves plants (both legumes and non-legumes) and specific diazotrophs (rhizobia and *Frankia*). During this symbiotic relationship, a niche and carbon molecules are provided to the microorganisms by the plant in exchange for nitrogen fixation (Schwember et al., 2019). The symbiotic nitrogen fixation is the most efficient fixing system which approximately fixes 170–300 kg of nitrogen per hectare per year. Symbiotic diazotrophs are dominant in the soil environment, where they arrive inside the root of the plant and form a nodule on the plant where the symbiotic dialogue takes place (Tu et al., 2016). This process is controlled by several genes like *nod*, *nif*, and *fix* genes. The main function of the nodule is to produce an environment that restrains oxygen-free flow and triggers the nitrogen fixation process (Oldroyd, 2013).

#### *Rhizobium*-Legume Symbiosis

Rhizobia are classical legume symbionts belonging to Betaproteobacteria and Alphaproteobacteria. They form an effective nodule mainly on the root and in a few cases on the stem of the legume host with few exceptions of non-legumes (i.e., *Parasponia*). *Parasponia* nodules have a tissue organization and ontogeny different from that of legumes but very similar to that of actinorhizal nodules (Pawlowski and Bisseling, 1996). Some legumes belonging to the genera *Neptunia*, *Aeschynomene*, and *Sesbania* bear nodules on both roots and stems. The rhizobial species are distributed among 18 genera of different families including Methylobacteriaceae, Rhizobiaceae, Bradyrhizobiaceae, Phyllobacteriaceae, Brucellaceae, Hyphomicrobiaceae, Burkholderiaceae, and Xanthobacteraceae (Mousavi, 2016; De Lajudie et al., 2019). Some genera (e.g., *Azorhizobium*, *Pararhizobium Allorhizobium*, *Neorhizobium*, *Sinorhizobium*, and *Mesorhizobium*) are known as promiscuous hosts while others (e.g., *Rhizobium*) are highly specific.

#### *Frankia*-Non-legume Symbiosis

Nitrogen fixation in the nodules of non-legumes usually takes place by the symbiotic association with *Frankia*. *Frankia* is a filamentous, Gram-positive actinomycete that forms a symbiotic relationship with plant species of 25 genera belonging to eight different families (220 species) of dicotyledonous plants including Betulaceae, Casuarinaceae, Myricaceae, Elaeagnaceae, Rhamnaceae, Rosaceae, Coriariaceae, and Datisticaceae (Normand et al., 2014). Most of these nitrogen-fixing non-legumes are found in poor, sandy soils low in nitrogen. The common examples are alder, bayberry, sweet fern, sweet gale, buffalo berry, bitterbrush, pine, and olives. Their ability to fix nitrogen is a significant factor in their survival under conditions that would be inhospitable to ordinary plants. There are two ways through which *Frankia* forms an association with actinorhizal plants; i.e., the intracellular and intercellular penetration (Carvalho et al., 2014). It has different types of cells, e.g., vesicles and spores, located on the sporangia and the vegetative hyphae. Vesicles are the actual cells that are formed under limited N conditions and contain enzymes responsible for N fixation.

### Cyanobacteria

Nonsymbiotic fixation of nitrogen involves fixation through heterotrophic or autotrophic organisms or by free-living diazotrophs called cyanobacteria. Cyanobacteria are the Gram-negative oxygenic photo-autotrophs that developed during the Precambrian period and reformed the anoxic earth’s atmosphere into oxic, which is appropriate for the process of oxygenic metabolism (Kaushik et al., 2017). Cyanobacteria have great agricultural importance. In freshwater and marine systems, they are the key N_2_ fixers which provide a great source of fixed nitrogen in the marine ecosystem in the world’s oceans. In terrestrial environments including rainforests and deserts, they abundantly grow and are involved in nitrogen fixation. Because of their special adaptations like nitrogen fixation and desiccation resistance ability, they can survive in harsh conditions. In modern agriculture, cyanobacterial mats have been used as biofertilizers as they provide more than 70% of the nitrogen fixed to the agricultural systems (Benavides et al., 2013; Berthelot et al., 2015). In the Baltic Sea, *A. flos aquae* fixes 75 percent nitrogen (Klawonn et al., 2016).

Under aerobic and anaerobic conditions, they can fix nitrogen in structures called cyanobacterial mats besides the progression of molecular H_2_. Cyanobacteria especially blue-green algae are the key players in the regulation of nitrogen and carbon cycling. These also have a positive effect on the inhibition of different diseases of plants through the population of host-pathogen and herbicides (Smith and Crews, 2014). Heterocystous cyanobacteria (e.g., *Nostoc*, *Anabaena*, and *Aulosira*) develop associations with roots (Prasanna et al., 2013). The nitrogen fixation capability is restricted not only to heterocystous cyanobacteria, but there are many non-heterocystous cyanobacteria which are filamentous and unicellular (e.g., *Aphanothece*, *Oscillatoria*, *Gloeothece*, *Gloeocapsa*, and *Plectonema*) which also fix nitrogen significantly.

## The Detail of Deep-Down Symbiotic BNF Dialogue

**Figure 3:**
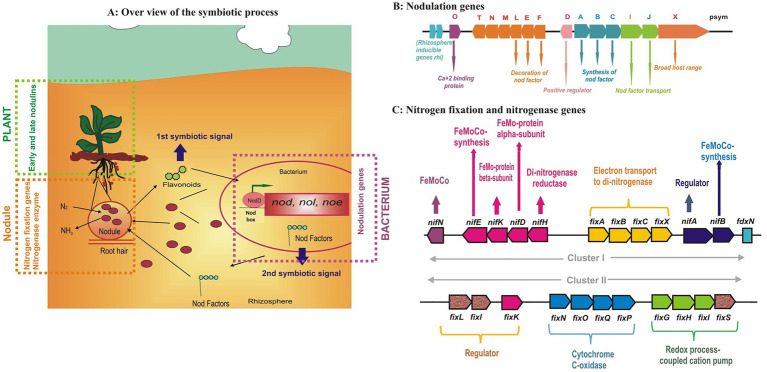
The overall process of *Rhizobium*-legume symbiosis showing the locks and keys of the symbiotic specificity of **(A)** nodulation genes **(B)** and details of nitrogen fixation genes **(C)** present in the rhizobia (figures drawn from the available literature from different sources).

The overall symbiotic process is a highly specific and sophisticated exchange of signals (Figure 3A) between both the partners involved; “the *macrosymbiont* and the *microsymbiont*.” The **first *symbiotic* signal** comes from the host in the form of “Flavonoids” which induce the expression of different genes (*nod*, *noe*, *nol*, and others) in the rhizobia within the vicinity of the root. The *nod* genes encode the synthesis of Nod factors decorated with host-specific modifications which act as the **second symbiotic signals**. Nod factors are released in the rhizosphere where they serve as ***the first rhizobial determinant of host specificity*** (Spaink, 2002). The Nod factor triggers several responses such as ion changes, calcium oscillations, and gene expression. Rhizobia are attracted to host plants by chemotaxis and attach themselves to the root hair with the help of exo/lipopolysaccharides. This attachment leads to root hair deformation and curling which provides a site for infection thread (IT) initiation (Nanda et al., 2010). Curling of the root hairs occurs due to the localized presence of Nod factor molecules. Simultaneously, root cortex cells are stimulated to reinitiate mitosis, leading to the formation of nodule primordia and the formation of a functional nodule starts.

Root hair curling results in the entrapping of attached bacteria within the deformation. Local lysis of the root hair cell wall is followed by invagination of the plant cell membrane. The process starts with the curling of root hairs, and a tube-like structure called an infection thread (IT) is formed through which the bacteria enter the plant. Infection threads are plant-derived structures originating from plasma membrane invagination accompanied by external deposition of the cell wall material. The IT grows inwardly toward the base of the root hair and the dividing cells in the nodule primordium. Intracellular infection is facilitated by the cell division in cortical cells. During IT development, rhizobial surface polysaccharides (lipopolysaccharides, exopolysaccharides, capsular polysaccharides, and cyclic glucans) interact with the host plant. Successful symbiosis depends on their correct composition, making them ***the second rhizobial determinant of host specificity*** (Perret et al., 2000). Bacteria reach the nodule primordium infecting several primordium cells and are released into the host cytoplasm by a process resembling endocytosis. During this release, the bacteria become surrounded by a plant membrane, and a symbiosome is formed. When the symbiosome divides, the infected cells become fully occupied with the microsymbiont. In actinorhizal symbioses and also in some legumes, bacteria are not released from the infection threads; rather, infected cells are filled with branching infection threads containing the microsymbionts, which is now called bacteroid. These bacteroids are surrounded by the plant membrane “peribacteriodal membrane” which forms a symbiotic interface with the host.

After infection, nodules are developed from the root cortical cells which are mitotically reactivated to form the nodule primordium (Mus et al., 2016) where thousands of bacteroids are present. Nodules are mainly of two types: “indeterminate” and “determinate.” The indeterminate nodules originate from cell divisions in the inner cortex and have a persistent meristem at their apex, and a clear “*spatial developmental gradient*” is formed from the distal meristem to the proximal root attachment site where these developmental zones can be characterized by the expression of specific plant genes. The determinate nodules originate from cell divisions in the outer cortex and do not have a persistent meristem, because the meristem of the nodule ceases to divide at an early stage of development and undergoes “*temporal developmental stages*” which are similar in the whole nodule. Legume nodules have a stem-like anatomy with peripheral vascular bundles and infected cells in the central tissue, while actinorhizal nodules have a root-like anatomy and develop from primordia formed in the pericycle.

The early stages of the symbiosis are controlled by highly specific chemical signals; the later stages by the expression of certain bacterial genes. During nodule morphogenesis, decreasing oxygen concentrations are maintained by the combined effects of specialized plant cells acting as an oxygen diffusion barrier and an abundant nodulin: the leghemoglobin. During this dramatic physiological switch, rhizobia initiate the expression of both nitrogen fixation genes and genes encoding a high-affinity terminal oxidase.

During nitrogen fixation, cyanobacteria usually differentiate into heterocyst which is the particular N_2_-fixing cells at consistent intervals of every 10–20 vegetative cells (Hilton et al., 2016). A nitrogen-fixing cyanobacterium (*Cylindrospermopsis raciborskii*) which lacks a *patL* ortholog at each end separates the trichome by almost a hundred vegetative cells and serves as a special site for the biosynthesis of nifH and activation of nitrogenase enzymes (Plominsky et al., 2013). A unicellular, photoheterotrophic bacteria (Candidatus *Atelocyanobacterium thalassa*) which lives with the genus *Braarudosphaera* (prymnesiophyte), the host usually attains fixed nitrogen from Candidatus *Atelocyanobacterium thalassa*. This heterotrophic nitrogen-fixing cyanobacterium has a Gamma A *nifH* phylotype, which is a dominant characteristic of a large aquatic proteobacterial *nifH* group.

### Nitrogenase Enzyme Complex

Fixation is the process in which nitrogen from the atmosphere moves into the soil. The symbiotic and associative N_2_ bacteria reduce the dinitrogen to ammonia using the nitrogenase enzyme. There is sufficient evidence showing the horizontal transfer of genes among the different prokaryotic species, for example green sulfur bacteria, proteobacteria, methanobacteria, and cyanobacteria (Ivleva et al., 2016). The nitrogenase enzyme consists of two metalloproteins (Figures 3B,C), (i) molybdenum-iron (MoFe) and (ii) Fe protein, which are named according to their metal composition. The molybdenum-iron protein, encoded by *nifD* and *nifK*, is a heterotetramer (α_2_β_2_) where actual binding and reduction of N_2_ take place. The Fe protein is a homodimer (α_2_) that is encoded by the *nifH* gene and responsible for providing electrons to the MoFe protein. These genes are part of the same operon and frequently appear as *nifHDK* (Dixon and Kahn, 2004). Besides, several genes such as *nifBEN* genes play a role in the biosynthesis of the iron-molybdenum cofactor of dinitrogenase, *nifA*, which is a regulatory gene, and *nifF* and *nifJ* genes encoding electron transport proteins are also present in the core operons. Thus, nitrogen fixation is regulated by nif regulon which is a set of various operons comprising genes encoding nitrogenases, proteins involved in electron transfer, and regulatory genes (Shin et al., 2016).

In addition to the molybdenum-iron nitrogenase system, two alternative vanadium and iron-only nitrogenases have been identified in *Azotobacter vinelandii* and *Rhodobacter capsulatus* that contain vanadium and iron at their active sites (Mus et al., 2018). These enzymes comprise VnfHDK and AnfHDK subunits that are homologous to the NifHDK subunits of Mo nitrogenase but only expressed under Mo-depleting conditions. Moreover, these nitrogenases contain either vanadium-iron or iron-iron cofactors at their active sites as well as additional components like VnfG and AnfG subunits with unknown function (Dixon and Kahn, 2004).

The BNF requires a reductant (flavodoxin, ferredoxin, or sodium dithionite) which delivers electrons for reduction of N_2_. In principle, six electrons are required for the reduction of N_2_ to NH_3_, but this process is also coupled with the generation of 1 mol of H_2_ (Newton, 2007). Therefore, in most of the diazotrophs, eight high-potential electrons are provided by reduced ferredoxin. The transfer of electrons from reductase to nitrogenase is also coupled with ATP hydrolysis by reductase.

### Nitrogen Assimilation and Metabolism

The prime assimilation of nitrogen through plants comprises of the utilization of a diverse kind of NO_3_^−^ or NH_4_^+^ (inorganic N), based on the availability of nitrogen, adaptation, and species of plants. The accumulation and sufficient assimilation of the secondary ammonia group (NH_4_^+^) also take place in the plants toward re-assimilating the ammonium ion which usually produces endogenously for different processes such as biosynthesis of phenylpropanoid, photorespiration, and amino acid catabolism in the plants (Betti et al., 2014).

The ammonia produced in legume nodules crosses the bacteroid membranes *via* diffusion and is taken up by the plant cell as NH_4_^+^ and/or NH_3_. To ensure the loss of ammonia *via* recovery by a bacterium from a plant cell, the bacterial NH_4_^+^ transporter AmtB is repressed in bacteroids which transports NH_4_^+^ in the opposite direction (Mus et al., 2016). The ammonia is then protonated in the acidic environment of the symbiosome and crosses the membrane through an NH_3_ channel and/or cation channel for K, Na, and NH_4_^+^ transport and subsequently assimilated into organic form in the cytoplasm (Day et al., 2001). The ammonia is assimilated into amino acids glutamine and glutamate through the action of enzymes such as glutamine synthetase (GS) and glutamate synthase which are produced during nodule development. The enzymes interact with different membrane transporters for efficient assimilation of ammonia; for instance, the cytosolic GS involved in the assimilation of ammonia to glutamine physically interact with nodulin 26, which can transport NH_3_ (Masalkar et al., 2010). Glutamine and glutamate act as nitrogen donors for the biosynthesis of essential amino acids and serve to translocate organic nitrogen to various parts in legumes as well as non-legumes.

Initially, glutamine and glutamate are used for the biosynthesis of aspartate and asparagine. Aspartate is an important part of the malate-aspartate shuttle that is involved in the translocation of electrons from mitochondria and chloroplast into the cytoplasm. It is a precursor for two main pathways: (i) synthesis of asparagine and (ii) synthesis of aspartate-derived amino acids such as lysine, methionine, isoleucine, and threonine (Xu et al., 2012). Asparagine is an important nitrogen storage and transport compound in legumes and non-legume plants and synthesized through the transfer of the amide group of glutamine to aspartate *via* the action of asparagine synthetase (Lomelino et al., 2017). Asparagine is catabolized to form ammonia through the asparaginase enzyme, and the produced ammonia is subsequently re-assimilated through the glutamate synthase cycle. The essential amino acids of the aspartate family appear to be synthesized in the chloroplast; however, the methylation of homocysteine which converts it into methionine has been shown to take place in the cytoplasm. Different amino acids are used to produce different proteins which constitute about 80% of plant N. Moreover, the purines which are components of nucleic acids contain nitrogen and are derived from two amide-N of glutamine, one amino-N of aspartate, and one amino-N of glycine. Similarly, pyrimidines are derived from aspartate and carbamoyl phosphate. The N of carbamoyl phosphate comes from the amide-N of glutamine *via* a reaction by carbamoyl phosphate synthetase with 2 ATP.

### Termination of the Symbiotic Dialogue (BNF)

Usually, the nodules have a limited lifespan and the symbiotic process is terminated gradually. Nodule senescence can occur at various developmental stages of the legume or may be triggered by some environmental stresses. It is commonly believed that after infection and differentiation, bacteroids of indeterminate nodules are terminally differentiated and are unable to return to a free-living state, while bacteroids of determinate nodules are thought to retain the capacity for free-living growth and can undergo a reverse differentiation process upon nodule senescence (Dixon and Kahn, 2004). It is, therefore, hypothesized that polyhydroxybutyrate (PHB) accumulation by bacteroids in the determinate nodules and by undifferentiated cells in the infection thread of indeterminate nodules may function to give the rhizobial cells a competitive advantage when released into the soil after nodule senescence.

## Regulation and Plant Growth During BNF

BNF is an extremely complex biological process known to be very sensitive to environmental conditions. The development of advanced molecular biology and next-generation sequencing technologies has shown us details of basic machinery and regulatory networks during BNF. The basic mechanism and machinery are similar to those already described in rhizobia-legume symbiosis. Diazotrophs fix atmospheric nitrogen, which improves the nitrogen contents, and are found within the roots and in the phyllosphere of various C3 and C4 crops (Li et al., 2019). In addition to providing fixed-N, diazotrophs also improve the N uptake from the soil which may be due to a general increase in the root surface area (Islam et al., 2013) as a positive correlation has been demonstrated between N uptake and root architecture. Evidence shows that diazotrophic bacteria exhibit several other traits that are considered important for plant development and yield. Production of phytohormone, siderophores, HCN, and antibiotics; solubilization of phosphorus, zinc, and potassium; calcite degradation; and 1-aminocyclopropane-1-carboxylic acid (ACC) deaminase activity are some of the growth-promoting mechanisms exhibited by associative and symbiotic diazotrophs (Hakim et al., 2020).

Plant growth is a complex phenomenon. Plants themselves continuously produce and secrete compounds, including organic/amino acids, sugars, phenolics, and other secondary metabolites into their surroundings for the selective assemblage of those microbes which help out the plants through multiple ways on practical grounds (Ali et al., 2020). On the other side, there is always a competition between the soil microbes for the successful colonization of the roots, and in most cases, microbes exhibiting high affinity for the roots and having multiple traits are favored by the plants. The PGP activities of diazotrophs have a well-documented effect on overall plant growth and yield in natural environments as well as experimental settings (Dobbelaere et al., 2003). Studies also demonstrate their role in stress alleviation under drought, salt and heavy metal stress (Valentine et al., 2018), restriction of the phytopathogens (Rosenblueth and Martínez-Romero, 2006), and increased bioaccumulation of metals from the soils to the biomass of plants (Ullah et al., 2015). Moreover, cold-adapted diazotrophs have also shown their potential to alleviate cold stress (Suyal et al., 2014, 2019; Kumar et al., 2020). Compared to synthetic fertilizers (N) which provide a single source of nutrients, diazotrophs serve as a multipurpose fertilizer and plant growth stimulator. Important plant-beneficial traits of diazotrophs important for BNF are discussed in detail in the following section.

### Role of Phytohormones

Phytohormone production by symbiotic and associative diazotrophs is the major mechanism promoting the growth of the host plant. Many plant hormones/growth regulators (auxins, gibberellin, cytokinin) are produced by these bacteria, and this ability is widespread (>80% inhabiting the rhizosphere) in the microbes. These hormones are important regulators of plant development, regulating different processes involved in root proliferation, early plant growth, root and shoot growth, plant elongation, etc. The secondary roots and root hairs are the preferred colonization sites for these diazotrophs. Rhizodeposition also takes place on the junctions of primary roots and rootlets for better and stable attachment to initiate nitrogen fixation and other cellular processes (Naqqash et al., 2020). Therefore, microbes act on the initiation of secondary roots and root hairs, cell division, and elongation in the roots. The bacterial association also regulates the plant’s inherent ability of hormone production, thus improving plant health (Carvalho et al., 2014). Microbial association and phytohormone production improve root growth and the root surface area, which facilitate the plant to harbor maximum sources of soil nutrients. A few examples of diazotrophs that produce phytohormones and their role in plant growth and development are described in Table 2.

**Table 2:** List of few diazotrophs with multi-trait characters important for the regulation of BNF and plant growth during BNF.

Diazotrophs	PGPR Traits	Effect on plant	Plant	Ref
*Azoarcus*,	Production of phytohormones -Indole-3-acetic acid (IAA), N2-fixation Gibberellins	Improvement in the lateral roots and root hairs in terms of number, length, weight, and volume significant improvement in root initiation, surface area, fine roots, fresh weight, and dry mass	Potato Rice Sugarcane Grasses wheat	Baca and Elmerich, 2007; Miyauchi et al., 2008; Naher et al., 2009; dos Santos et al., 2019a,b; Naqqash et al., 2020;
*Azospirillum*,				
*Azotobacter*,				
*Brevundimonas*,				
*Gluconacetobacter*,				
*Herbaspirillum*,				
*Klebsiella* etc.				
*Sinorhizobium meliloti*	Cytokinin			Kisiala et al., 2013
*Bradyrhizobium*	AHLs, N2 fixation	Increased root hair formation in seedlings	Soybean, wheat	Ali et al., 2016
*Rhizobium*,	Improved P acquisition, N2 fixation	Increase the uptake of nutrients to aerial parts of the plants	*Cicer arietinum Cassia absus* *Vigna trilobata Sesbania sesban* Lettuce, wheat Rice, mustard	Halder et al., 1990; Narula et al., 2002 ; Baldani and Baldani, 2005; Sridevi and Mallaiah, 2009; Estrada et al., 2013; Tahir et al., 2013; Nosrati et al., 2014; Verma et al., 2020
*Bradyrhizobium*,				
*Azospirillum*,				
*Azotobacter*,				
*Burkholderia*,				
*Herbaspirillum*,				
*Rhizobium*,	N2 fixation	Improved Zn in the plant and grain	Wheat, cotton Maize, tomato Red pepper, Mung bean Soybean, Lettuce, green gram	Islam et al., 2013; Naz et al., 2016; Shaikh and Saraf, 2017; Kallimath and Patil, 2018; Kumawat et al., 2019; Tagele et al., 2019; Velmourougane et al., 2019; Verma et al., 2020
*Bradyrhizobium*,				
*Azospirillum*,				
*Azotobacter*,				
*Burkholderia*,				
*Herbaspirillum*,				
*Trabusiella*,				
*Serratia*, *Klebsiella*				
*Azotobacter* sp.	N2 fixation, P solubilization, auxins, siderophores	Improved plant growth and mineral nutrition especially Fe	Maize, tomato	
*R. rhizogenes*	HCN. Antibiotics, siderophores	Control the pathogen Induce systemic resistance		Chauhan et al., 2012; Das et al. 2017; Zhang et al., 2018; Kerr and Bullard, 2020; Kong et al., 2020
*Pantoea*,		Reduced disease incidence of *Ralstonia solanacearum* and increased root and shoot dry weight		
*Burkholderia*,				
*Arthrobacter*				
			
		Suppress the growth of *Fusarium oxysporum* and *Rhizoctonia solani* and significantly reduce the disease incidence		
*Azospirillum* spp.,	Antibiotics and antifungal compounds			
*Azotobacter*			Cotton and rice	
*chroococcum*, and				
*Gluconacetobacter diazotrophicus*				
*Achromobacter xylosoxidans*	Acc-deaminase	Increase plant root system, improve plant growth, and tolerate salinity stress		Karthikeyan et al., 2012
*Bacillus* sp.	ACC deaminase, IAA, P solubilization	Improved growth, induced plant response for defense enzymes, chlorophyll, proline, soluble sugars	Maize	Misra and Chauhan, 2020
	EPS			
*Azorhizobium*,	IAA, P solubilization	Increase in nitrogen uptake and improved root and shoot growth	Tomato red pepper	Bashan et al., 2004; Islam et al., 2013
*Azospirillum*,				
*Bacillus*,				
*Burkholderia*,				
*Herbaspirillum*, and				
*Paenibacillus*				
Actinobacteria mostly *Curtobacterium* spp. and *Microbacterium* spp.	IAA, P solubilization, stress tolerance	Salinity tolerance, high *nifH* expression in stem and root	*Salicornia europaea* L.	Hrynkiewicz et al., 2019
*Pseudomonas* spp.				
*Dyadobacter* sp.	Clod-tolerant, nitrogen-fixing	Improved growth and soil N	Finger miller and pulses	Kumar et al., 2018

### Role of Soil Nutrients

The BNF is a complex exchange of nutrients between the partners. Both organisms change their metabolic routine to fine-tune and accommodate the BNF which is monitored and regulated by both partners. Apart from C and N, several other compounds are made available to symbiotic microbes especially in the case of symbiotic (endophytic) diazotrophs which rely on the host for their functioning. Nutrient application (e.g., P) improves the overall soil microbial N and soil N fixation rates (Reed et al., 2007) because P is essential for the metabolism of both the host and the microsymbiont. Nodules are strong sinks for P, and limited P decreases the BNF (Yelenik et al., 2013). The studies also support the fact that high plant P contents are required for the development of symbiotic BNF in legumes (Divito and Sadras, 2014). Iron is an essential component of nitrogenases as well as leghemoglobin and is usually transferred across the symbiotic membrane. Likewise, sulfur is also an essential component of the nitrogenase enzyme and transported through the symbiotic membrane (Krusell et al., 2005). Besides, diazotrophs required cobalt, molybdenum, and vanadium (Bellenger et al., 2008) and deficiency of any of these elements result in the development of nonfunctional nodules.

Multi-trait associative or symbiotic diazotrophs (enlisted in Table 2) solubilize the unavailable compounds of different nutrients (e.g., P, Zn, Ca, Fe, etc.) and convert them into plant-available forms, thus increasing their uptake to aerial parts of the plants (Dobbelaere et al., 2003) and having an overall positive impact on plant growth and BNF (Piceno and Lovell, 2000). Moreover, the stimulation of BNF in legumes has been reported by the addition of P-solubilizing arbuscular mycorrhiza (Pueschel et al., 2017).

### Oxygen Regulation

The BNF enzyme “nitrogenase” is extremely sensitive to oxygen and rapidly inactivated in the presence of oxygen. Legumes, as well as non-legumes like *Parasponia* and actinorhizal plants, maintain microaerobic conditions through various mechanisms using various physiological strategies: (i) anaerobic growth to avoid high oxygen level, (ii) increased rate of respiration involving a specialized cytochrome, (iii) conformational protection under oxygen stress conditions which involve the association of a FeSII protein with nitrogenase thus temporarily inactivating the enzyme (Maier and Moshiri, 2000), and (iv) oxygen diffusion barriers through the production of an alginate capsule (Sabra et al., 2000). The microaerobic conditions trigger a signaling cascade in rhizobia which involves the activation of FixL which is an oxygen sensor protein. FixL induces the expression of transcriptional activator FixJ, which in turn activates nifA and fixK, subsequently inducing the expression of various proteins involved in BNF. Furthermore, the rhizobia express a heme-copper cbb3-type oxidase with high oxygen affinity. The nitrogenase enzyme of *Streptomyces thermoautotrophicus* is unaffected by the presence of oxygen. The enzyme contains manganese superoxide dismutase as nitrogenase reductase which shows no homology with *nifH* (Ribbe et al., 1997). Moreover, this Mo nitrogenase requires significantly lower energy as compared to standard nitrogenases.

In nitrogen-fixing cyanobacteria, different ecological, biochemical, and morphological adaptations are established to reduce the oxygen-associated complications. The paradox of oxygenic photosynthesis diazotrophs is resolved by the segregation of oxygen-sensitive machinery for the fixation of nitrogen in heterocysts. Oxygenic photosynthesis is separated from nitrogen fixation spatially or temporally in non-heterocystous cyanobacteria. In temporal separation, BNF happens during the shadowy period and photosynthesis during the light period, while in the spatial separation, the dominant non-photosynthetic cells develop and are involved in BNF and the upper green cells become photosynthetic (Issa et al., 2014).

### BNF Under Stress

Several of the environmental conditions limit the growth and activity of the N_2_-fixing plant as well as the diazotrophs. BNF is mostly suppressed under different biotic and abiotic factors, e.g., pathogen, salt, drought, acidity, alkalinity, heavy metals, fertilizers, etc. (Zahran, 1999). These stress factors suppress the growth and BNF potential of most of the diazotrophs. Salt and drought stress inhibit the initial steps of BNF, i.e., root hair curling and infection thread development. A high stress level reduces the nodule number and size, or complete inhibition of nodulation, and nitrogen fixation is reduced due to a reduction in nodule respiration. Similarly, high temperature (above 35–40°C) affects root hair infection, bacteroid differentiation, nodule structure, and functioning (reviewed by Zahran 1999). Diazotrophs are highly sensitive to fertilizer-managing practices which reduce the diversity, abundance, and the structure of diazotroph community (Reardon et al., 2014; Wang et al., 2017; Xiao et al., 2020). T-RFLP profiling of N_2_-fixing bacterial communities in the rhizosphere was found decreased at higher N-fertilization rates, showing a negative influence of fertilizer on the abundance of diazotrophs (Meng et al., 2012). Metatranscriptomic analysis revealed a diverse set of diazotrophs associated with switchgrass roots with enrichment of a subset of diazotrophs in roots of N-deficient plants that exhibited the highest nitrogen fixation rates. Higher levels of N fertilization resulted in lower levels of associative nitrogen fixation (ANF) in the roots where at the maximum level (180 kg N/Ha) it essentially abolished the ANF (Bahulikar et al., 2020).

Many of the diazotrophs have ecologically evolved to manage these stresses by different mechanisms. Some of the diazotrophs are enlisted in Table 2. For adaptation under salinity, two distinct classes of osmoprotectants exist in diazotrophs: one such as proline, glycine betaine, and glutamate, and another which acts as a chemical mediator such as ectoine (Zahran, 1999). Drought-tolerant and heat-tolerant diazotrophs produce different enzymes and proteins and produce 1-aminocyclopropane-1-carboxylate (ACC) deaminase, which lowers the concentration of ethylene in plant roots by catabolizing ACC, a precursor of ethylene. Consequently, the plant can cope with a variety of ethylene-inducing stresses and proliferate its root system. Diazotrophs can inhibit the growth of a diverse range of plant pathogens causing various diseases by producing antibiotics, hydrogen cyanide (HCN), and siderophores or inducing systemic resistance in the plants (Das et al., 2017; Ali et al., 2020). So, the development of diazotrophic inoculants with dual attributes of nitrogen fixation and antagonism against phytopathogens can contribute to increased plant growth and productivity.

Other diazotrophs, e.g., Cyanobacteria, are usually recognized from an extended evolutionary past due to their distinctive traits such as high yield of biomass, production of beneficial biofuels and by-products, and oxygenic photosynthesis, improving the fertility of the soil and saline soil reclamation.

## Cropping System Modifications to Upscale BNF for an Ecosystem Benefit

The review and potentials, opportunities, and limiting factors of BNF suggest that many priority issues be addressed for researchers and policymakers. The involvement of diazotrophic microorganisms in the BNF process is one of the important agricultural practices to improve crop yield as well as environmental quality. Diazotrophs and host plants positively interact with each other, which help in reducing the demand of N fertilizers (Ai’shah et al., 2009). The total annual terrestrial inputs of N from BNF have been estimated from 139 to 175 million tons of N where symbiotic associations account for 25–30% (35–44 million tons of N) and permanent pasture accounts for the other 30% (45 million tons of N; Paul, 2000). Although the accuracy of the estimates of N inputs through BNF may be open to question, its relative importance in cropping and pasture system can be improved to substitute a part of the million tons of N fertilizer applied to the agriculture system annually (dos Santos et al., 2017).

### Bioinoculants

BNF is no doubt an environment-friendly approach for the improvement of soil fertility and the production of crops (Bashan et al., 2004). Maximum benefits from BNF can be obtained by diazotrophic inoculation to different cropping systems. Several reports illustrate (few enlisted in Table 3) that the application of diazotrophs completely or partially eliminates the need for chemical N fertilizers. Inoculation of different rhizobia on respective hosts increases plant growth comparable to N fertilizer treatment (Jensen et al., 2020). An experiment with a combined application of N-^15^-labeled nitrogen and diazotrophs on pine reveals that 30% less nitrogen fertilizer is required when diazotrophic bacteria were applied (Bal and Chanway, 2012). In some cases, inoculated plants are even better as the bacteria contain multiple properties that stimulate a plethora of plant growth parameters rather than the sole N nutrition. These results and many more signify the fact that the diazotrophs partially fulfill the N needs of the plants and their inoculation at reduced N fertilizer gives similar growth and yield responses as those grown with full N fertilizers. This natural and eco-friendly crop fertilization will reduce the input of synthetic fertilizers to agriculture and reduce overall GHG emissions in the atmosphere.

**Table 3:** List of few diazotrophs’ inoculation under field conditions for BNF under reduced application of N fertilizer.

Diazotrophs	N details of the experiment	Effect on plant	Plant	Ref
*Herbaspirillum*, *G. diazotrophicus* *Nitrospirillum* *Paraburkholderia*	Inoculation	Produce plant regulators which enhance the activity and expression of different enzymes involved in the plant metabolism of nitrogen	Sugarcane	dos Santos et al., 2019a,b
*Herbaspirillum* and *G. diazotrophicus*	Inoculation	Increase in the activity of genes associated with reduction and uptake of nitrate in inoculated plants		Nogueira et al., 2001
*H. seropedicae*, *A. brasilense*,	33% N fertilizer + inoculation	Increased a number of different maize metabolites, showing specie specific plant-bacterial interaction and functional nitrogenase activity Height and chlorophyll content were increased	Maize	Kifle and Laing, 2016; Brusamarello-Santos et al., 2017
*A. brasilense*	Inoculation	Reducing the need of nitrogen fertilizers	Maize	Longhini et al., 2016
*A. brasilense*	Inter cropping with xaraes grass	Minimum production of dry mass and increased crude protein content due to N coverage fertilization, up to the dose of 120 kg ha^−1^ N	Maize	Longhini et al., 2017
*Azospirillum* sp.	50% of N fertilizer + inoculation	Growth of plant especially tiller count was the same in low condition and inoculation as in the standard condition of nitrogen	Rice	Sasaki et al. 2010
*Herbaspirillum* sp	Zero N + inoculation	Same growth as with 100% N fertilizer	Rice	de Souza et al., 2013
*Burkholderia vietnamiensis*	Inoculation only	Grain yield was increased 29% in comparison to those plants where 100 kg N per hectare fertilizer was added Increases the seed germination rate and vigor Increased N accumulation through enhanced N absorption ability and root morphology	Rice	da Silva Araújo et al., 2013; Kifle and Laing, 2016; Shinjo et al., 2020
*Azospirillum brasilense*, *Bacillus cereus, Acinetobacter calcoaceticus*	50% N + inoculation	Growth performance, nitrogen uptake, and biomass yield were similar to full fertilizer	Sugarcane	Hossain et al., 2020
Diazotrophic bacteria	Zero N + inoculation	Comparable yield increase to that with 120 kg ha^−1^ N fertilization	Sugarcane RB72454	Schultz et al., 2014
PGPB	Applied in combination with reduced N fertilizer	Significant increase in the relative chlorophyll index, tiller units, yield, total nitrogen uptake, and nutrient concentration of total nitrogen by combining nitrogen fertilizers with PGPB	*Zuri Guinea* grass	Carvalho et al., 2020
		Enhances the uptake of nitrogen, calcium, manganese, iron, and ammonia Increases the relative chlorophyll index, ultimately increasing the yield		de Lima et al., 2020
*Bradyrhizobium* sp.	Without any fertilizer + inoculation	Similar growth and grain yield as full N fertilizer	Soybean	Kaschuk et al., 2016

The main thing to consider is to have a clear understanding of the factors affecting BNF under field conditions for improving inoculum quality and ultimately strengthening the capacities of farmers. The need for improvising the BNF, particularly by fertilizers or in the symbiosis of N-fixing microbes, must be addressed internationally. The best tool for reducing the yield gap is the integration of BNF to plant-breeding programs and to develop better formulation technologies for bioinoculants. Furthermore, a significant percentage of the bioinoculants should be subsidized for small farmers like the chemical fertilizers.

### Ecological Intensification and Crop Diversification

With ecological intensification (EI), it is possible to increase food production by redesigning agricultural lands in such a way that both biodiversity and ecological processes are not hampered. EI increases crop production per area by exploiting ecological processes that assist beneficial organisms by diversifying croplands and enhances profitability by minimizing the need for costly processes. The agricultural matrix can be made both more permeable and habitable to biodiversity by planting a variety of crops into landscapes which will help in conserving biodiversity gradually. EI practices facilitate the habitation and resources for colonizing beneficial organisms (e.g., microorganisms or soil invertebrates) that promote crop growth. Beneficial soil microorganisms cycle and/or supply nutrients to plants, while the invertebrates decompose organic matter and aerate soils and prey on crop pests (Kremen, 2020). Analysis of the effect of management (ecological intensive vs. conventional intensive) and comparison of the diazotrophic communities in both systems show that ecological intensive management promoted a beneficial N-related microbial community composition involved in N-cycling processes (Lori et al., 2020).

Crop diversification (CD) has proved to be beneficial to improve crop production as well as to reduce market and production risks which are caused by tremendous price fluctuations and uncertain water availability, respectively. These practices have reduced the risk of pests, drought, and diseases, hence increasing the overall productivity of the system without compromising the yield (Manjunatha et al., 2013). Many models and meta-analyses have reported that crop diversification is positively correlated with increased profitability and productivity (Letourneau et al., 2011; Davis et al., 2012; Avasthe et al., 2020). The CD practices include cultivation of diverse crops (Renard and Tilman, 2019), mixed cropping (Bedoussac et al., 2015), rotation of diverse crops (Reckling et al., 2016), and cultivation of region-adapted crop varieties (Yang et al., 2019). Crop rotations and cropping sequence diversity are essential factors influencing bacterial assemblages and species diversity because different crops produce different root exudates which attract different bacterial population. Soil fertility management, crop protection, and crop rotation have all been shown to exert significant effects on the soil microbial communities present in the agricultural soils. The effect of fertilization rate and crop rotation analysis on cyanobacterial diazotrophs community structure showed that rice-mustard-moong (RMM) crop rotation is more suitable for cyanobacterial nitrogen fixation than rice-wheat-maize rotation. Furthermore, nitrogenase activity was found higher in the cropped plots than fallow plots. The low fertility coupled with RMM rotation was found to be best suited for promoting nitrogen fixation by cyanobacteria to supply the rice plants (Jha et al., 2001).

### Legume Integration

Legumes are regarded as agricultural and ecological wonders because they utilize the normal sources of nitrogen available in the air through BNF and improve soil fertility. The BNF ability of different legumes is variable; e.g., *Leucaena* 325, Lucerne 250, pigeonpea 220, cowpea 210, mung bean 200, soybean 110, groundnut 100, chickpea 102, and common bean 50 kg N Ha^−1^ (Serraj, 2004). The introduction of legumes into the pastures has been seen as the best strategy to improve nitrogen nutrition of grasses (between 75 and 97 kg N ha^−1^ in 97 days growth of *Stylosanthes guianensis*; Viera-Vargas et al., 1995). It was observed that the denitrification rate was lower in the unfertilized non-legume-cultivated areas in contrast to the legume-cultivated areas. This is an indication that by growing legumes and mineralization of the lower carbon-nitrogen ratio of its residues, the availability of soil nitrogen will increase (Zilli et al., 2020).

The addition of any legume crops in the current cropping system will have lasting benefits along with the enhanced crop productivity and profitability (Choudhury and Kennedy, 2004). The legumes in the cropping system will improve the abundance of diazotrophs (Joshi et al., 2007; Zou et al., 2020). Similarly, the addition of *Pisum sativum* in the agriculture system enhanced the abundance of free-living diazotrophs in Eastern Oregon, USA (Reardon et al., 2014). Rahman and Chima (2016) established a diagnostic model for crop diversification and revealed a significant correlation between diversifying a system into multiple crops and profit generation. So, crop diversity magnanimously contributes to overall sustainable agriculture.

### Technological Development

Available information indicates that the current application of BNF at a large scale is generally constrained by the poor effectiveness and competitiveness of the technology compared to other alternatives for soil fertility management. This requires a concerted effort for improving the efficiency and stability of the technology, especially in a stressful environment. Different organic fertilizers, e.g., livestock manure and crop residues, contribute to the reestablishment of the diazotroph population (Liao et al., 2018). These properties of fertilization on rhizospheric diazotroph may be direct, due to the increased availability of several nutrients, for example phosphorus, carbon-nitrogen ratio, availability of nitrogen, and soil organic carbon, and indirect by different soil features, such as pH of the soil (Wang et al., 2016, 2017). Furthermore, the diazotrophs in the environment are prone to different harsh conditions of weather which may affect their stability and population. Investment in the BNF will ensure economic profitability, energy efficiency, environmental quality, and agricultural sustainability.

Nanotechnology holds great promise for upgrading the conventional fertilizer industry. Many nano-formulations of chemical fertilizers are being tested in the agriculture system that have low leaching losses, are not easily volatilized, and do not cause any deterioration to land quality. Nano-based products, e.g., nano-sensors, nano-pesticides, nano-fertilizers, nano-films, nano-magnets, and nano-filters, are available in the market which will change the agricultural production if applied on larger scale. Nanotechnology has materialized the concept of bacterial encapsulation where single and composite polymers are used as carriers of beneficial plant microorganisms to increase the performance and consistency of bioinoculants and minimize costs and the effect on the environment (Pacheco-Aguirre et al., 2017). Due to their smaller size, nanoparticles provide a large surface area for the diazotrophs to grow and enable plants to uptake nutrients slowly and sustainably as needed by plants. The coating shields diazotrophs from unfavorable environments and control a gradual release in the soil.

A new frontier would be to combine the benefits of nano-formulations of chemical fertilizers with the diazotrophs to formulate hybrid fertilizers. These fertilizers may be coated with biodegradable polymers that degrade slowly in the rhizosphere. It will function as a single multipurpose fertilizer that serves the purpose of biological nitrogen fixation and as a slow-releasing chemical fertilizer. It will minimize the chemical fertilizer input into the agriculture system, thus saving billions of dollars to the economy and the ecosystem. On the other hand, it will boost the fertilizer industry to a new hybrid fertilizer industry that will have more acceptability at the farmer level and national level and in the scientific community because it will be safer for future generations and the environment. This approach has been explained well in the Graphical Abstract.

## Summary and Implications

Nitrogen is the major element required by the crops and is critical for growth yield. It has played a phenomenal role in the success of the Green Revolution but at the cost of adverse impacts on the quality of the overall environment. Chemical fertilizers are not available to everyone everywhere, and they cannot completely maintain the equilibrium of the ecosystem. The potential economic and environmental benefits of BNF favor investing in the diazotroph technology. Although BNF is already making a large contribution in total N-fixed globally, in agriculture, its use and benefits can be maximized by adding legumes in the cropping systems. Legumes have a large scope (species >3,000) with open prospects for developing ecologically sustainable and economically viable agricultural cropping systems. Field data of diazotroph inoculation to legumes and non-legumes presented in this review show that BNF can substitute 30–50% of the fertilizer-N demand of different crops. This not only saves the farmer input (by saving fertilizer) but also improves overall returns (by decreasing pollution and increased sustainability). Despite a lot of research and investment in BNF, problems like old-fashioned technology, inadequate availability, nonuniformity of product and results, and climate issues render the wide-scale adaptation by the farmers. To harness the maximum potential of BNF, technology should be improved and refined using the advancements made in the 21st century. Developing nano-hybrid formulations will improve not only efficacy, application, stability, and shelf life but also product uniformity. It will revolutionize the whole biofertilizer industry. Governments should encourage the private sector to come forward and develop a nano-hybrid inoculant industry. Farmer participation should be increased in technology development and evaluation. A holistic approach and better consideration of the market, agricultural policies, legislation, and investment of private sector will support the BNF to maximize the benefits for the ecosystem and beyond.

## Author Contributions

AI conceived the review, formulated the layout, and edited the final version. SH collected and reviewed the literature regarding nitrogenase enzyme. MT collected and reviewed the literature regarding legumes. MN collected and reviewed the literature regarding symbiotic specificity. IL collected and reviewed the literature regarding nitrogen usage in agriculture. UG collected and reviewed the literature regarding rhizobium-legume symbiosis. MKH collected and reviewed the literature regarding symbiotic diazotrophs. MS collected and reviewed the literature regarding multiple diazotrophic properties. MH collected and reviewed the literature regarding health concerns of excessive N-application. MA and AF formulated the tables, made the figures and formatted the reference list. All authors contributed to the article and approved the submitted version.

### Conflict of Interest

The authors declare that the research was conducted in the absence of any commercial or financial relationships that could be construed as a potential conflict of interest.
